# A New Sign in UV Dermoscopy for Diagnosing Pthiriasis: The Glowing Crab Louse Sign

**DOI:** 10.7759/cureus.91664

**Published:** 2025-09-05

**Authors:** Aimane Zaim, Zakia Douhi, Meryem Soughi, Fatima Zahra Mernissi

**Affiliations:** 1 Department of Dermatology, Hassan II University Hospital, Sidi Mohamed Ben Abdellah University, Fez, MAR

**Keywords:** blue fluorescence, dermoscopy, glowing crab louse sign, pthirus pubis, ultraviolet dermoscopy

## Abstract

*Pthirus pubis*, commonly known as the pubic louse, is an obligate ectoparasite causing sexually transmitted infestation, typically presenting with intense pruritus in the pubic and genital regions. Atypical localizations, including eyelashes, axillae, chest, and scalp hair, have been described. Accurate diagnosis can be challenging in individuals with dense hair or low parasite burden. We report a case of a 27-year-old man presenting with two weeks of severe pubic pruritus. He reported multiple episodes of unprotected sexual intercourse, with no systemic illness or immunosuppression. Clinical examination was hindered by dense pubic hair. Dermoscopy revealed multiple *P. pubis* parasites and nits, exhibiting a characteristic “scorpion-like” appearance. Ultraviolet-induced fluorescence dermoscopy highlighted a novel feature, with bright blue fluorescence emitted by the entire parasite, termed the “glowing crab louse sign”. The patient was successfully treated with a 10% dimeticone-based topical agent. Symptoms resolved within one week, and follow-up dermoscopy confirmed complete eradication. This report introduces the “glowing crab louse sign” as a novel dermoscopic marker for diagnosing pthiriasis. It may aid clinicians in challenging cases, enhance diagnostic accuracy, and could be incorporated into artificial intelligence-assisted diagnostic models. Further studies are needed to validate this feature and explore its underlying fluorescence mechanism.

## Introduction

*Pthirus pubis*, commonly known as the pubic louse, is an obligate ectoparasite. The World Health Organization classifies pubic louse infestation as a sexually transmitted disease. Although global prevalence has declined markedly over the past decades, mainly due to changes in grooming habits, sporadic cases are still encountered in clinical practice [1]. The infestation typically presents with intense pruritus in the pubic and genital regions, although atypical localizations, including eyelashes, axillae, chest, and even scalp hair, have also been reported [2].

Accurate and timely diagnosis is essential to ensure its effective management and prevent further transmission. However, detection may be challenging in individuals with dense terminal hair or low parasite burden. Entomodermoscopy has emerged as a valuable non-invasive diagnostic tool, enabling rapid in vivo identification of parasites and their nits [3]. Innovations such as UV-induced fluorescence dermoscopy (UVFD) have further enhanced diagnostic capabilities for ectoparasitic infestations, particularly in scabies and pediculosis, by revealing unique fluorescence patterns [4,5].

In this report, we present a case of pubic pruritus in which UV dermoscopy facilitated the identification of *P. pubis*, highlighting a previously undescribed feature, the “glowing crab louse sign”.

## Case presentation

We present the case of a 27-year-old man who had been experiencing intense, disruptive itching in the pubic region for two weeks. The patient reported multiple instances of unprotected sexual intercourse with different partners. No prior systemic diseases or immunosuppressive states were reported.

**Figure 1:**
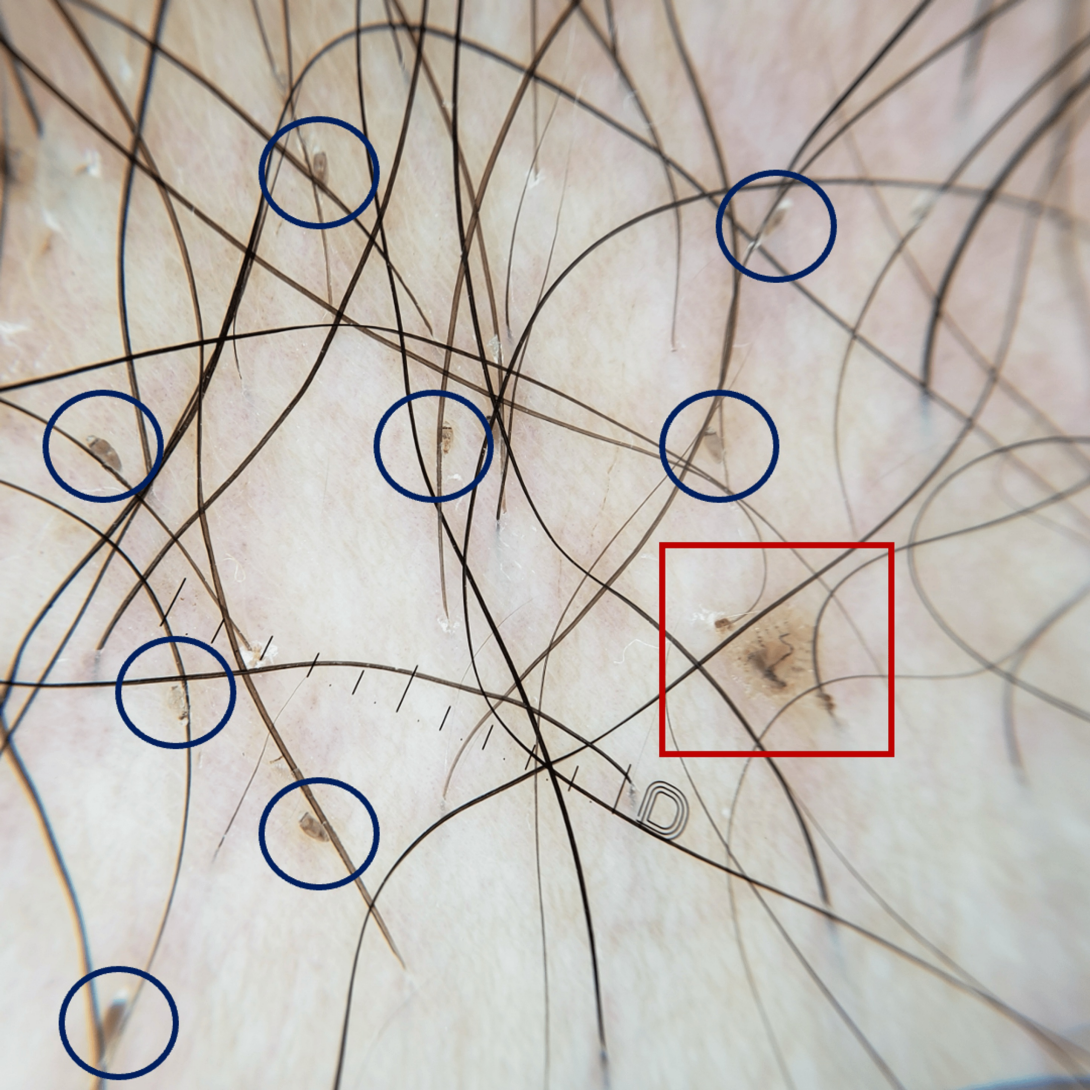
Dermoscopic image showing multiple nits (blue circles) and the characteristic scorpion-like appearance of Pthirus pubis (red square).

**Figure 2:**
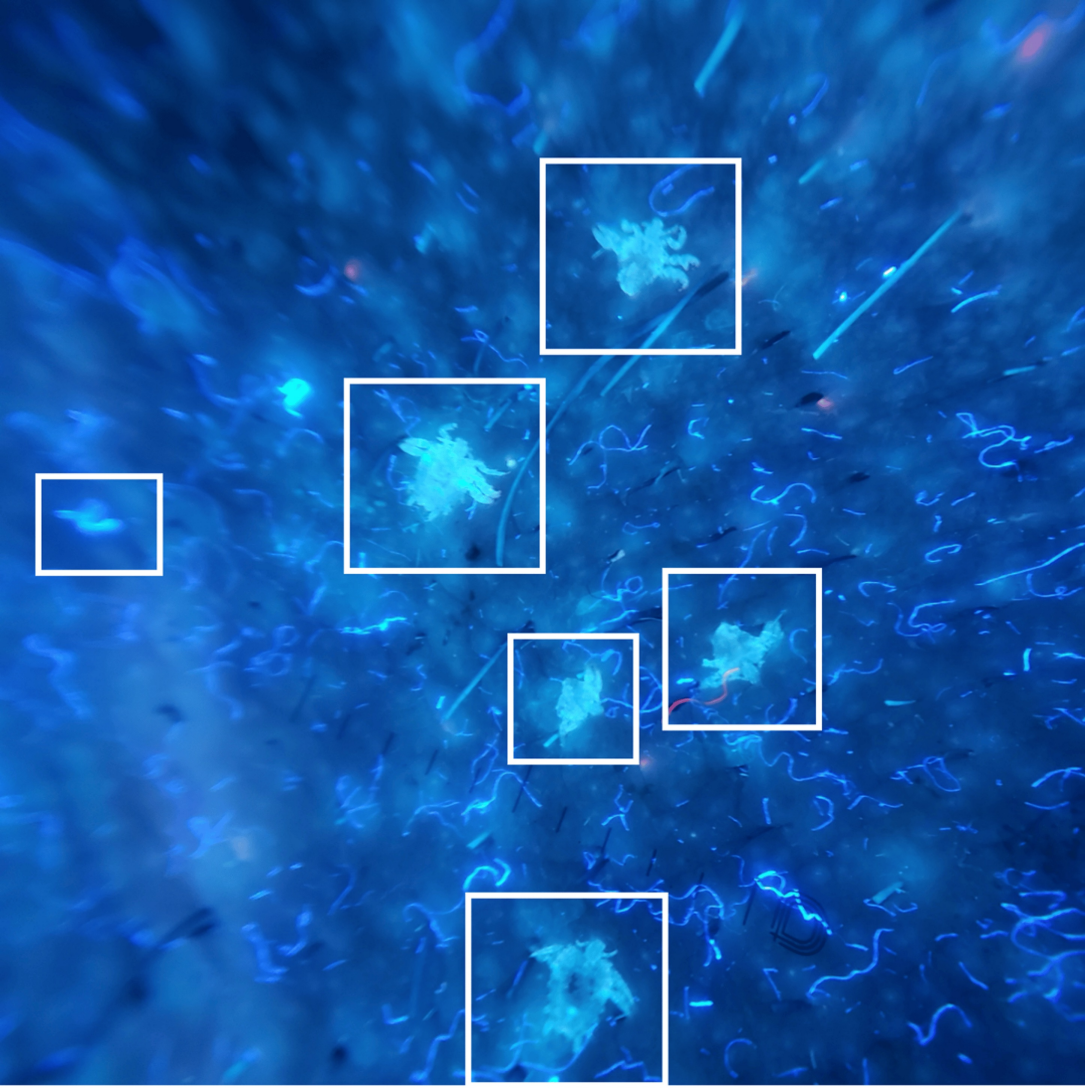
Blue fluorescence of the entire body of multiple Pthirus pubis under UV dermoscopy, demonstrating the "glowing crab louse sign" (white squares).

**Figure 3:**
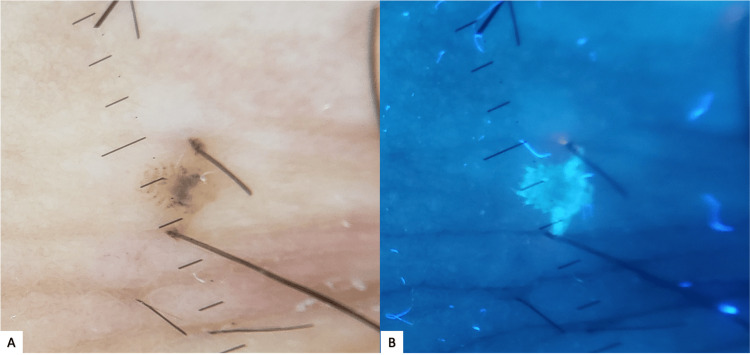
Dermoscopic images showing the scorpion-like appearance of Pthirus pubis (A), and the “glowing crab louse sign” under UV mode (B).

Clinical examination was challenging due to the patient’s excessive pubic hair. Dermoscopy revealed both nits and several *P. pubis* parasites firmly attached to pubic hairs, exhibiting a characteristic “scorpion-like“ appearance (Figure 1). Notably, UVFD highlighted a novel feature, with bright blue fluorescence emitted by the entire body of *P. pubis*, which we have termed the "glowing crab louse sign" (Figure 2). A comparative illustration juxtaposing polarized dermoscopy (Figure 3A) and UVFD (Figure 3B) highlights the contrast between the classical “scorpion-like” morphology and the newly described fluorescence feature.

The diagnosis of pubic lice was confirmed, and the patient was successfully treated with a 10% dimeticone-based topical agent. A full sexually transmitted infection screen was negative. Symptoms resolved within one week, and follow-up dermoscopy showed complete eradication of parasites.

## Discussion

Infestation by *P. pubis* (pthiriasis) remains an important, albeit increasingly uncommon, sexually transmitted parasitic disease. *P. pubis* is a permanent, blood-feeding ectoparasite that grasps pubic and perianal hair using its characteristic crab-like claws, making transmission primarily via close physical or sexual contact, although indirect spread via fomites (e.g., towels, bedding) can occur. Historically, prevalence ranged from 2% to 10%, but has declined sharply since the early 2000s, largely due to changes in personal grooming practices, particularly the widespread removal of pubic hair. As a result, documented cases are now rare, and the pubic louse may be nearing extinction in developed populations [1].

The parasite feeds by ingesting blood while injecting saliva and anticoagulants into the host, which can trigger allergic reactions and intense pruritus, often leading to scratching, erythema, papules, and secondary infections [6]. Occasionally, infestations may be asymptomatic or affect atypical sites, including the legs, abdomen, chest, axillae, back, eyelashes, and rarely the beard or scalp, thus complicating clinical recognition [2,6].

Traditional diagnosis relies on direct visualization of lice or nits, which may be difficult in patients with dense pubic hair or in early infestations. Bites can also leave small (<1 cm) gray-blue spots, known as maculae ceruleae [1].

Dermoscopy has proven to be a valuable tool in diagnosing pthiriasis [7], allowing for the direct visualization of *P. pubis*, which is larger and more flattened compared to *Pediculus capitis*, and firmly adheres to pubic hair while feeding. The parasite’s blood-filled intestines give its body a distinctive scorpion-like appearance [3]. Nits can also be detected in the pubic hair, providing further evidence of the infestation.

Recent advances in dermatoscopic techniques, particularly UVFD, have significantly improved the diagnostic accuracy for various cutaneous infestations. For instance, UVFD has been shown to be highly effective in diagnosing scabies, where the mite emits a bright green fluorescence, referred to as the "ball sign" [8,9]. Similarly, it has been applied to the diagnosis of pediculosis, revealing a distinctive tulip pattern, in which nits adhere to the hair shaft through its glue. This pattern allows differentiation of pediculosis from other hair shaft conditions, such as white Piedra, characterized by clear, soft, white nodules arranged in a "constellation pattern" along the hair shaft [5].

This is an original description of the blue fluorescence emitted by *P. pubis* under UV dermoscopy. The exact chromophore responsible for this phenomenon remains unidentified and warrants further investigation to explore its diagnostic and potential therapeutic implications.

## Conclusions

This report introduces the "glowing crab louse sign" as a new dermoscopic feature for diagnosing pthiriasis. This distinctive fluorescence pattern could be especially helpful for clinicians with less experience, particularly in challenging cases where the diagnosis is not immediately evident, thereby improving diagnostic accuracy. Additionally, it could be integrated into future artificial intelligence-assisted diagnostic models and used as a tool for monitoring treatment efficacy. Further studies are needed to confirm the reproducibility of this sign and to elucidate the biochemical mechanism underlying the observed fluorescence.
